# Oral manifestations of renal tubular acidosis associated with secondary rickets: case report

**DOI:** 10.1590/2175-8239-JBN-2018-0105

**Published:** 2018-09-06

**Authors:** Susilena Arouche Costa, Soraia de Fátima Carvalho Souza, Ana Margarida Melo Nunes

**Affiliations:** 1 Universidade Federal do Maranhão Programa de Pós-Graduação em Odontologia São LuísMA Brasil Universidade Federal do Maranhão, Programa de Pós-Graduação em Odontologia, São Luís, MA, Brasil.; 2 Universidade Federal do Maranhão Faculdade de Odontologia São LuísMA Brasil Universidade Federal do Maranhão, Faculdade de Odontologia, São Luís, MA, Brasil.

**Keywords:** Renal Tubular Acidosis, Dentistry, Oral Manifestations

## Abstract

This report describes the oral manifestations of renal tubular acidosis (RTA) associated with secondary rickets and discusses the biological plausibility of these findings. The characteristic electrolyte changes during RTA or genetic mutations that trigger RTA may be responsible for impaired amelogenesis, dental malocclusion, impacted teeth, and absent lamina dura. This report reinforces the possibility of an association between RTA and the oral manifestations described.

## INTRODUCTION

Renal tubular acidosis (RTA) is characterized by abnormalities in the transport mechanism of the distal and proximal tubules of the kidneys that lead to decreased HCO_3_ reabsorption or H^+^ excretion, resulting in metabolic acidosis, which may impair bone metabolism.^1^ These electrolyte imbalances may affect the amelogenesis mechanism^2^^-^6 and the craniofacial development and lead to dental malocclusion.^3^

Although the association between RTA and oral manifestations is biologically possible, there is no strong evidence to date to support this hypothesis. Therefore, the objective of this report was to describe the association between RTA and the oral manifestations and discuss the possible biological mechanisms underlying this association.

## CASE REPORT

R.E.L.S., a female patient aged 14 years and 5 months, was dissatisfied with the color of her teeth and sought the Dentistry Clinic in São Luís (Maranhão, Brazil). Her mother reported a history of preterm birth, jaundice at birth and a diagnosis of RTA (OMIM 179800) associated with secondary rickets at the age of 4 years. In addition, the patient had low weight (31 kg), short stature (1.35 m), and mild thinness (BMI = 17 kg/m^2^).

**Figure 1:**
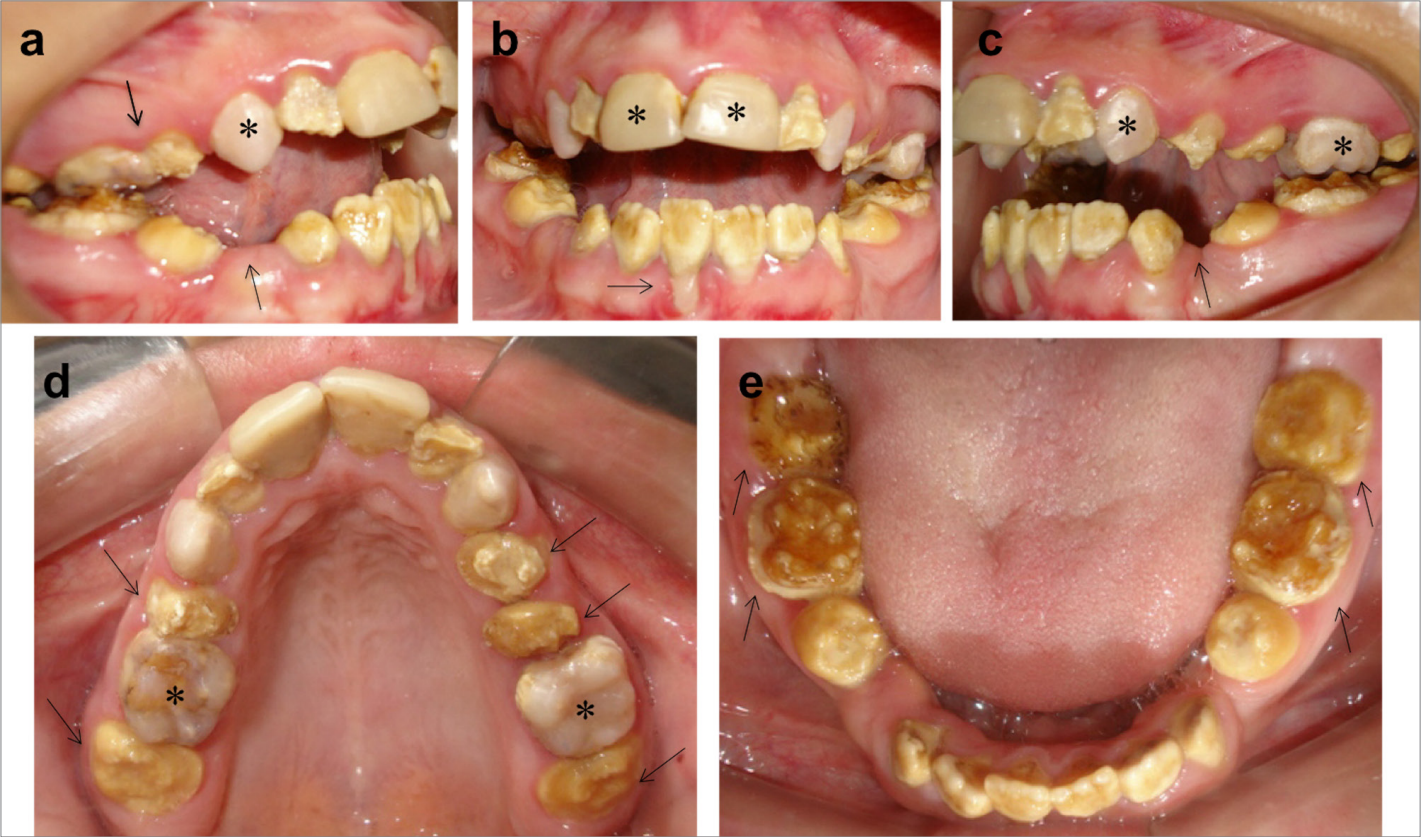
Oral examination showing permanent dentition with yellow-brownish teeth and rough tooth surface. (a) Lateral view of the right hemiarch showing the absence of teeth 15 and 44 (arrows). (b) Frontal view showing anterior open bite and dehiscence with gingival recession on tooth 41 (arrow). (c) Lateral view of the left hemiarch showing absence of tooth 34 (arrow). (d) Occlusal view of the maxilla showing loss of enamel with severe dentin erosion in the posterior teeth (arrows). (e) Occlusal view of the mandible showing loss of enamel with severe dentin erosion (arrows) and composite resin restorations on teeth 16, 13, 11, 21, 23, and 26 (asterisks).

Clinical oral examination revealed yellow-brownish permanent teeth and loss of enamel in the posterior teeth with severe dentin erosion. Teeth 16, 13, 11, 21, 23, and 26 were restored (Figure 1a, b, c and d), whereas teeth 15, 34, and 44 were absent (Figure 1a and c). The patient had poor oral hygiene, generalized gingivitis and dehiscence with gingival recession only in tooth 41 (Figure 1b). In addition, she presented with both mouth and nose breathing, anterior open-bite malocclusion, absence of lip sealing, and no history of deleterious oral habits (Figure 1b).

**Figure 2:**
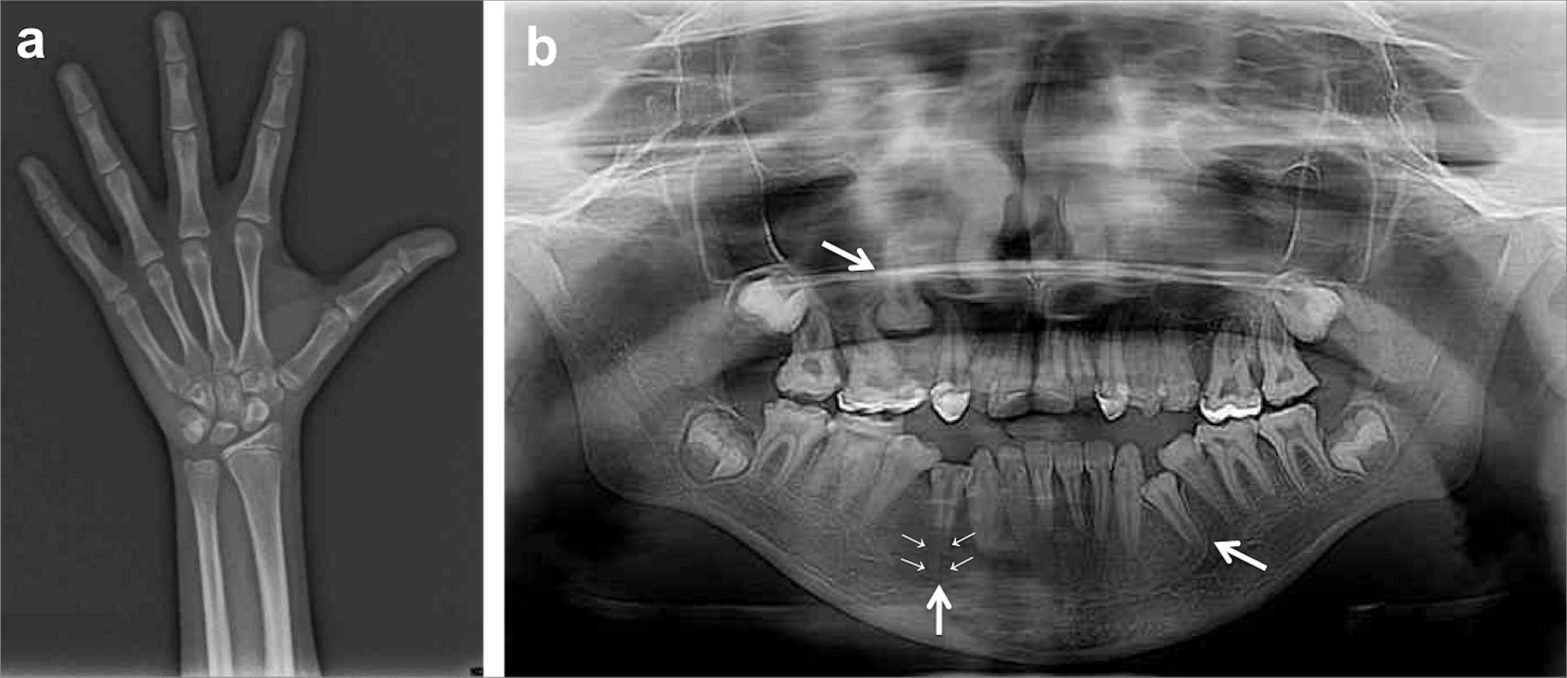
(a) Hand-wrist radiograph. (b) Panoramic radiograph showing loss of contrast between the enamel and dentin, particularly in the posterior teeth. Larger arrows indicate the unerupted teeth 15, 34, and 44, and smaller arrows indicate the absence of lamina dura in tooth 44.

Despite the diagnosis of secondary rickets during early infancy, her bone age was 13 years and 6 months according to hand-wrist radiography performed using the method of Greulich and Pyle (Figure 2a). Panoramic radiograph showed decreased radiopacity of the enamel and loss of contrast between the enamel and dentin in several teeth, suggesting hypoplastic amelogenesis imperfecta (HAI). Teeth 15, 34, and 44, which were not observed on visual inspection, remained unerupted and exhibited changes in radiodensity between the enamel and dentin, which were more evident in tooth 44 that lacked the lamina dura (Figure 2b).

## DISCUSSION

In this case report, oral manifestations including HAI, anterior open-bite malocclusion, impacted teeth, and absent lamina dura, were observed in a teenage patient with RTA.

Because this condition is rare, there is no strong evidence of an association between RTA and HAI. However, some studies on mice have indicated that a possible explanation for the occurrence of HAI in individuals with RTA is the mutation in the NBCe1-A locus of *SLC4A4*.^5^^,^^7^ This locus is responsible for regulating pH in the kidneys^1^ and ameloblasts. However, despite the ability of ameloblasts to dynamically regulate the pH of the enamel matrix,^5^ it has been demonstrated that the systemic pH of mice contributes to the enamel phenotype.^7^ Therefore, these findings suggested that RTA would be a predictor of HAI in the permanent dentition.

Anterior open bite has been described in individuals with RTA.^3^ However, in the present case, rickets due to electrolyte changes in RTA was possibly responsible for the alterations in the craniofacial development, dental malocclusion and retention of teeth 15, 34, and 44, possibly because of the lack of space for tooth eruption.

Dentin sensitivity in individuals with HAI may adversely affect oral health and eating habits, thereby limiting mastication. Therefore, severe dentin erosion with the consequent increase in dentin sensitivity may aggravate the nutritional status in such individuals.^8^ Thus, early dental intervention is essential to minimize the effects of RTA in the oral cavity.

In summary, our findings corroborate those of previous studies and reinforce the possibility of an association between RTA and the oral manifestations described. In addition, we recommend the early intervention of the dentist in order to plan preventive strategies to improve the quality of life for individuals with RTA.
